# Genetic abnormalities detected in ependymomas by comparative genomic hybridisation

**DOI:** 10.1038/sj.bjc.6600180

**Published:** 2002-03-18

**Authors:** M Carter, J Nicholson, F Ross, J Crolla, R Allibone, V Balaji, R Perry, D Walker, R Gilbertson, D Ellison

**Affiliations:** Department of Neurosurgery, Southampton General Hospital, Southampton, UK; Department of Child Health, Southampton General Hospital, Southampton, UK; Department of Cellular Pathology, Southampton General Hospital, Southampton, UK; Wessex Regional Genetics Laboratory, Salisbury, UK; Department of Paediatric Oncology, Addenbrookes Hospital, Cambridge, UK; Department of Histopathology, Queen's Medical Centre, Nottingham, UK; Department of Child Health, Queen's Medical Centre, Nottingham, UK; Department of Child Health, Royal Victoria Infirmary, Newcastle upon Tyne, UK; Department of Developmental Neurobiology, St Jude Children's Research Hospital, Memphis, Tennessee, USA; Department of Neuropathology, Newcastle General Hospital, Newcastle upon Tyne, UK

**Keywords:** ependymoma, CGH, gain 1q, loss 22, intermediate ploidy

## Abstract

Using comparative genomic hybridisation, we have analysed genetic imbalance in a series of 86 ependymomas from children and adults. Tumours were derived from intracranial and spinal sites, and classified histologically as classic, anaplastic or myxopapillary. Ependymomas showing a balanced profile were significantly (*P*<0.0005) more frequent in children than adults. Profiles suggesting intermediate ploidy were common (44% of all tumours), and found more often (*P*<0.0005) in tumours from adults and the spinal region. Loss of 22q was the most common specific abnormality, occurring in 50% of spinal (medullary) ependymomas and 26% of tumours overall. Genetic profiles combining loss of 22q with other specific abnormalities – gain of 1q, loss of 6q, loss of 10q/10, loss of 13, loss of 14q/14 – varied according to site and histology. In particular, we showed that classic ependymomas from within the cranium and spine have distinct genetic profiles. Classic and anaplastic ependymomas with gain of 1q tended to occur in the posterior fossa of children and to behave aggressively. Our extensive data on ependymomas demonstrate significant associations between genetic aberrations and clinicopathological variables, and represent a starting point for further biological and clinical studies.

*British Journal of Cancer* (2002) **86**, 929–939. DOI: 10.1038/sj/bjc/6600180 www.bjcancer.com

© 2002 Cancer Research UK

## 

Ependymomas are gliomas that exhibit degrees of ependymal differentiation (Burger and Scheithauer, 1995; Ellison, 1998). Typically, they develop in relation to the ventricular system and the cauda equina. They account for only 4–8% of gliomas, but are the third most common central nervous system (CNS) tumour of childhood, after astrocytomas and medulloblastomas. About 90% of paediatric ependymomas are intracranial, but in adults most are intraspinal (Gilles *et al*, 1995; Hamilton and Pollack, 1997; Bouffet *et al*, 1998; Gjerris *et al*, 1998; Wiestler *et al*, 2000). The World Health Organisation (WHO) pathological classification (Wiestler *et al*, 2000) recognises classic (WHO grade 2), anaplastic (grade 3) and myxopapillary (grade 1) variants, plus the subependymoma (grade 1). Classic and anaplastic variants mainly occur as posterior fossa tumours in children and young adults, and myxopapillary tumours nearly always present in the cauda equina of adults (Hamilton and Pollack, 1997; Ellison, 1998; Packer, 2000).

In children with intracranial ependymoma, event-free survival after 5 years is less than 50% (Bouffet *et al*, 1998; Robertson *et al*, 1998; Horn *et al*, 1999; Packer, 2000; Grill *et al*, 2001). Various factors have been reported to influence prognosis, though clinical research in this area has produced many conflicting results. However, gross surgical resection and the use of radiotherapy have been consistently associated with enhanced event-free and overall survival (Nazar *et al*, 1990; Sutton *et al*, 1990; Healey *et al*, 1991; Vanuytsel *et al*, 1992; Ferrante *et al*, 1994; Pollack *et al*, 1995; Bouffet *et al*, 1998; Robertson *et al*, 1998; Horn *et al*, 1999; Grill *et al*, 2001). Histological features of anaplasia, such as mitoses, microvascular proliferation and necrosis, serve as indicators of biological behaviour in other gliomas, including diffuse astrocytic tumours and oligodendrogliomas (Cohadon *et al*, 1985; Burger and Green, 1987; Ellison, 1998). However, the biological significance of these morphological features in ependymoma remains unclear; clinicopathological studies have provided conflicting evidence on the prognostic value of dividing ependymomas into classic (WHO grade 2) and anaplastic (grade 3) variants (Ross and Rubinstein, 1989; Sutton *et al*, 1990; Schiffer *et al*, 1991; Gerszten *et al*, 1996; Bouffet *et al*, 1998; Figarella-Branger *et al*, 2000).

With very few definite clinical or pathological markers of biological behaviour, the identification of genetic abnormalities responsible for the generation and maintenance of the malignant phenotype in ependymomas will be crucial, if there are improvements to be made in the management of patients with this disease. Not only may such defects serve as markers of aggressive disease, allowing more efficient use of existing therapies, but they may also suggest biological targets for novel therapeutic approaches. Cytogenetic studies have shown that chromosomal abnormalities are relatively common in ependymomas (Stratton *et al*, 1989; Neumann *et al*, 1993; Hamilton and Pollack, 1997; Mazewski *et al*, 1999; Vagner-Capodano *et al*, 1999). However, there are few large studies of genetic abnormalities in this disease, and their role in tumour behaviour therefore remains unclear.

We have employed comparative genomic hybridisation (CGH) to study tumours from a large population of patients with ependymoma. Specifically, our aims were to identify associations between patterns of genetic imbalance and the principal histopathological variants of ependymomas (classic/anaplastic and myxopapillary), which occur at distinct sites in adults and children. Such findings may enable future research to be targeted at genetic markers with potential prognostic significance.

## MATERIALS AND METHODS

### Tumour samples

Tumours (*n*=86) came from patients (*n*=77) treated in three regional UK neurosurgical units (Newcastle, Nottingham, Southampton, UK) between 1985 and 1999. Eight patients contributed more than one sample (seven patients – two samples, one patient – three samples) from consecutive resections of their tumour. In 16 other cases, the single sample analysed was not from the first tumour resection. In all three centres, a portion of each tumour was snap frozen and stored in liquid nitrogen, while the remainder was fixed in buffered formalin for histological examination. Frozen tumour samples were transported on dry ice to a single institution (Wessex Regional Genetics Laboratory) for CGH analysis.

Surgical resection had been the primary therapy in all cases, although most patients had received adjuvant chemotherapy and/or radiotherapy at some stage (Table 1

**Table 1:**
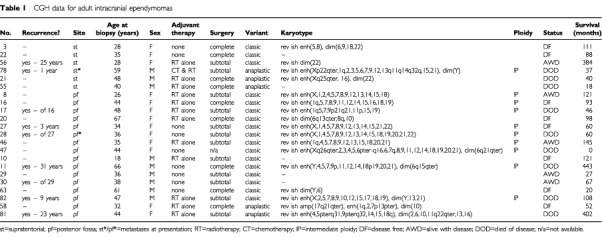
CGH data for adult intracranial ependymomas

). The male : female ratio was 1 : 1. Across the entire group, age at presentation ranged from 8 months to 69 years, with a median of 29 years (Table 1). Adults (median age: 39 years) contributed 58 samples, and children (<16 years; median age: 6 years) contributed 28 samples. The primary site for tumours was: supratentorial – 10 (13%), posterior fossa – 31 (40%), spinal cord – 14 (18%), cauda equina – 22 (29%). All tumours from children were intracranial, except one myxopapillary example, which presented in the filum terminale of a boy aged 15 years. No patients with spinal tumours had metastatic disease at presentation, but three intracranial tumours were found, during staging investigations, to be associated with metastases in the CSF pathways.

Histological assessment of each tumour was undertaken by two neuropathologists (R Allibone and D Ellison), using criteria from the WHO classification of central nervous system tumours (Wiestler *et al*, 2000); of a total of 86 tumours, 44 (51%) were designated classic ependymomas, 24 (28%) anaplastic and 18 (21%) myxopapillary. All frozen tumour samples for CGH analysis were examined histologically to confirm the neoplastic nature of at least 80% of the tissue.

### Comparative genomic hybridisation

CGH was performed on frozen tumour tissue using an indirect technique modified from those reported by Kallioniemi *et al* (1994) and our own group (Nicholson *et al*, 1999). Genomic DNA was labelled by nick translation with biotin, and sex-matched control DNA with digoxigenin (both from Roche Diagnostics Ltd). Probe mixtures containing 2 μg of labelled tumour and sex matched control DNA were hybridised with 50 μg Cot 1 DNA (Gibco–BRL) and 20 μg herring sperm carrier (Sigma) at 37°C for 3 days to sex-matched normal target lymphocyte metaphase spreads prepared in the Wessex Regional Genetics Laboratory. Biotin-labelled probes were detected with two rounds of avidin-FITC interspersed with one of biotinylated anti-avidin (Vector Laboratories) and digoxigenin-labelled probes were detected with mouse anti-digoxigenin, rabbit anti-mouse, and finally goat anti-rabbit TRITC (Sigma). Labelled detected metaphases were then mounted in antifade solution containing 1.5 μg ml^−1^ 4,6′-diamino-2-phenylindole (DAPI) counterstain (Vector laboratories). Each CGH experiment included a control hybridisation using normal DNA.

### Image capture and CGH analysis

Hybridisations were viewed under a fluorescence microscope and three colour images were captured by a cooled charged couple device camera (Photometrics), in conjunction with Macprobe 4.1 software (Perceptive Scientific International Ltd, Chester, UK). Metaphases were karyotyped and green : red fluorescence intensity ratios calculated along the length of each chromosome. Mean ratio profiles, together with profiles corresponding to ±1 standard deviation (s.d.), were constructed from 5–10 metaphases per tumour. Gains or losses of material in the tumour were inferred by deviation of the mean ratios beyond thresholds set at 1.15 and 0.85 respectively, providing the s.d. profile deviated to the same side of the midline. Selected cases with apparent imbalance on chromosomes 19 and/or 22 were chosen for CGH experiments with reverse labelling, because of concerns about artefacts in these regions. All abnormal results were confirmed by these experiments, using tumour DNA labelled with digoxigenin and controls with biotin. Gains associated with ratios greater than 1.5 were interpreted as amplifications.

### Fluorescence *in situ* hybridisation (FISH)

In a number of tumours the pattern of loss and gain was suggestive of intermediate ploidy (Rosenberg *et al*, 1997). This term is used where an increased copy number applies to some, but not all, chromosomes, and overall ploidy lies between one level and the next (usually diploidy and triploidy). Fluorescence *in situ* hybridisation (FISH) was performed on extracted nuclei from selected cases to confirm this. Loss/gain thresholds were then skewed to normalise for individual diploid chromosomes and the results interpreted with respect to the diploid state.

The FISH methodology allows an accurate and reproducible analysis of formalin fixed, paraffin wax embedded tumour material, and has been described in detail elsewhere (Nicholson *et al*, 2000). Briefly, cytospin preparations of nuclei were produced from sections (2–3×15 μm) of tumour. Digoxigenin-labelled or biotin-labelled plasmid probes to the centromeric regions of chromosomes 4, 6, 7, 8, 10, and 17 were applied singly or in pairs to preparations of nuclei. Probe hybridisation was conducted overnight at 37°C in a humidified chamber followed by stringency washes at 43°C using 0.1–0.5×SSC plus 30% formamide to remove non-specifically bound probe. Probes were visualised using a mixture of Texas red-labelled avidin and FITC-conjugated anti-digoxigenin antibody.

### Data analysis

Relationships among variables were assessed using standard statistical techniques: 2×2 contingency tables (Fisher's exact test), Log Rank analysis to produce Kaplan–Meier survival curves, and multivariate Cox analysis. Survival analysis of patients with spinal tumours was impossible because three of only five patients in the uncensored category had died in the immediate post-operative period.

## RESULTS

The results of CGH analysis are detailed by age/site in Tables 1, 2

**Table 2:**
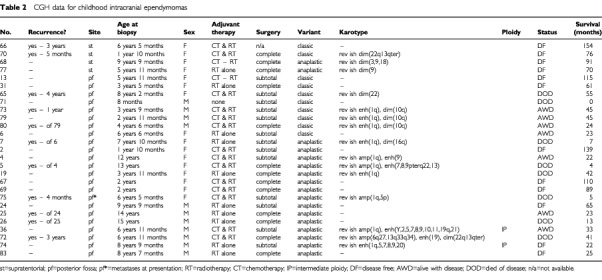
CGH data for childhood intracranial ependymomas

, 3

**Table 3:**
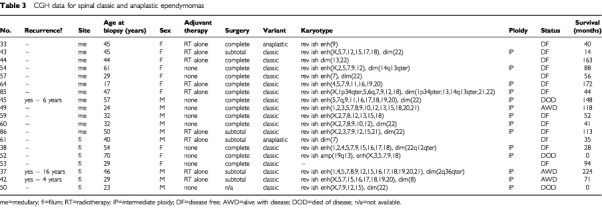
CGH data for spinal classic and anaplastic ependymomas

, 4

**Table 4:**
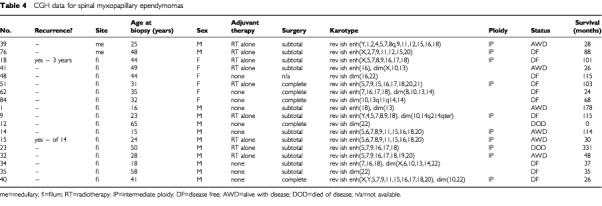
CGH data for spinal myxopapillary ependymomas

, alongside clinical parameters, and by histology in Figure 1

**Figure 1:**
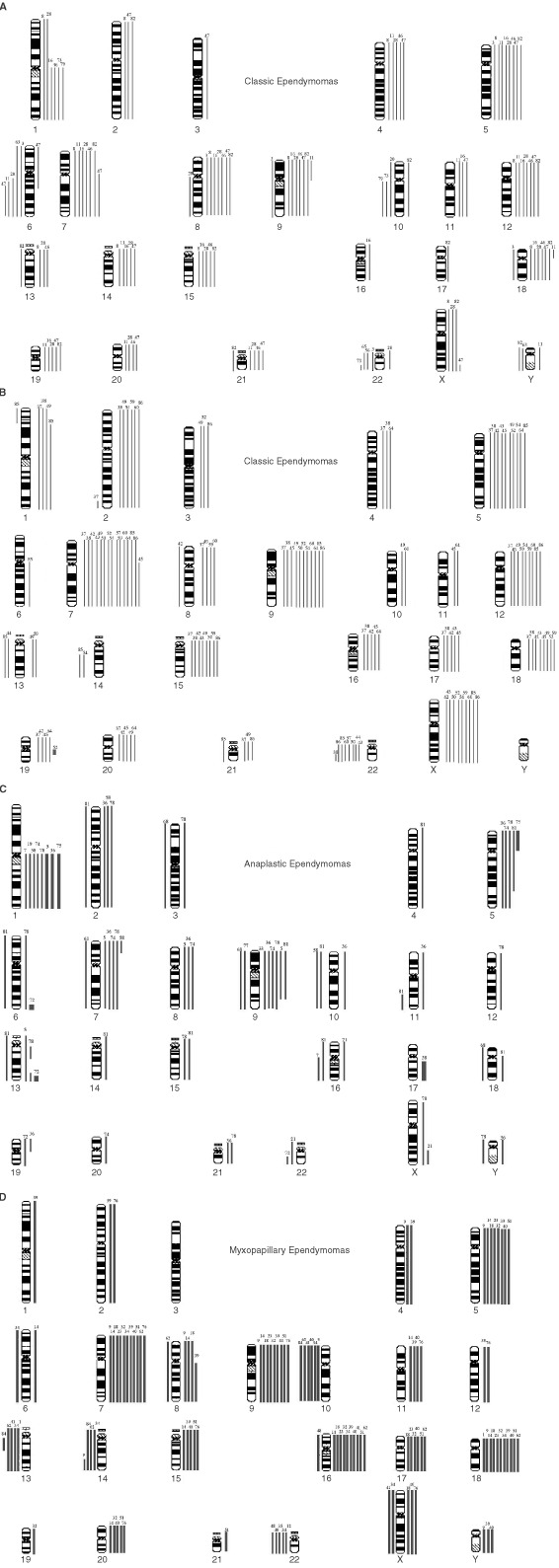
CGH ideograms divided according to histological variant/site: (**A**) Intracranial classic tumours; (**B**) Spinal classic tumours; (**C**) Anaplastic tumours; (**D**) Myxopapillary tumours. Loss and gain bars are on the left and right sides of each chromosome respectively.

. Balanced karyotypes were particularly prevalent among childhood tumours (*P*<0.0005), and were not found in myxopapillary variants. Ependymomas showing imbalance had a mean of six abnormalities. The progressive acquisition of genetic abnormalities was demonstrated by only one of eight ependymomas for which successive biopsies were available. This tumour acquired gain of 1q and loss of 16q, having shown a balanced profile at first surgery. This change accompanied progression of the tumour's histological features from classic to anaplastic.

**Figure 2:**
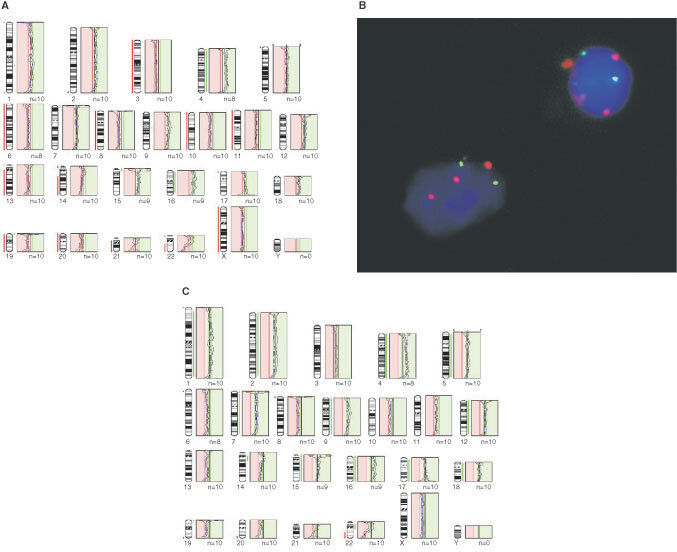
CGH profiles and FISH from case 38. Standard thresholds (**A**) imply a severely hypodiploid karyotype, but none of the profiles follows the mid-line. This is characteristic of intermediate ploidy cases, and FISH with centromere probes (**B**) confirms that there were two copies of chromosome 8 (green) in all cells and three copies of chromosome 17 (red) in many. Skewing the midline to the left to give a chromosome 8 profile midway between the gain and loss thresholds (i.e. normal) produces a profile (**C**) that implies gain of a large number of chromosomes, although distal 22q is still clearly lost. By this interpretation chromosomes 4 and 16 could have two extra copies compared to a single extra copy of the other gained chromosomes, or could be gained in all cells while a smaller proportion of cells have the other gains.

A high proportion of tumours (44%) demonstrated particular patterns of gain, and occasionally loss, across multiple chromosomes that were highly suggestive of intermediate ploidy. This interpretation was confirmed by FISH in a number of representative cases (Figure 2

). Certain chromosomes, 2/5/7/9/12/15/18/X, were commonly involved in these patterns. Such aberrations were more frequent in myxopapillary and classic tumours than in anaplastic variants (*P*=0.0199), in spinal relative to intracranial ependymomas (*P*<0.0005), and in tumours from adults *vs* children (*P*<0.0005).

The acquisition of certain genetic abnormalities: gain 1q, loss 6q, loss 10q/10, loss 13, loss14q/14 and loss 22 appeared distinct from the gains and losses of whole chromosomes that reflect intermediate ploidy, and the characteristics of tumours with these abnormalities were analysed. Loss of 22 was the commonest abnormality, detected in 20 tumours (26%). This change was found in all histological variants, though relatively less in the anaplastic tumours. However, loss of 22 was significantly (*P*=0.0154) associated with a spinal rather than an intracranial location. Gain of 1q and loss of 6q were particular features of classic/anaplastic ependymomas of the posterior fossa. Nearly all (11 of 13) tumours showing gain of 1q had come from the posterior fossa, and eight of the 13 were designated as anaplastic. Of all the posterior fossa anaplastic ependymomas with gain of 1q (*n*=8), all but one was from a child. All tumours with loss of 6q were from the posterior fossa. In contrast, seven of nine ependymomas showing loss of 13 and all showing loss of 14q/14 were spinal. Myxopapillary ependymomas were associated with both loss of 13 and loss of 14q/14. Loss of 10q/10 was present across histological variants, but significantly (*P*=0.0192) more often in myxopapillary tumours. All of the spinal tumours with losses on chromosome 10 were myxopapillary (*P*=0.006).

From data on the selected genetic abnormalities above, we tested the hypothesis that non-myxopapillary spinal ependymomas have different genetic profiles from those of myxopapillary and intracranial ependymomas. We compared the profiles of classic ependymomas from intracranial and spinal sites (Figure 1A,B), finding significant differences (*P*<0.0001). Both adults and children contributed intracranial tumours to this cohort (to ensure adequate numbers for the analysis), but all spinal tumours in this analysis were from adults (only one spinal tumour in the series was from a child). We also compared the profiles of classic plus anaplastic ependymomas from intracranial and spinal sites, again finding distinct profiles (*P*<0.0001). All tumours in this cohort were from adults. Finally, our analysis of spinal ependymomas from adult patients revealed significantly different profiles for classic and myxopapillary ependymomas (*P*<0.0001).

High level gain (CGH ratio >1.5 : 1) was infrequent, and mostly implied gain of several copies of the whole, or most of, a chromosome arm. It occurred in only six tumours, five of which were anaplastic (Table 1). Half of these tumours, showing gain of 1q, were from children. Single, focal high level gains were located at 6q27 and 13q33q34 in one child's tumour, and at 17q21qter and 19q13 in the remaining two tumours.

**Figure 3:**
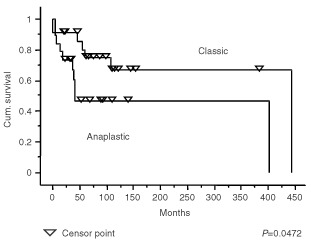
Kaplan–Meier plots showing a significant difference (*P*=0.0472) in the survival of patients with intracranial classic ependymomas and intracranial anaplastic ependymomas.

Because of the association between gain of 1q and anaplastic ependymomas, we examined the effect of histological diagnosis and gain of 1q on the survival of patients with intracranial tumours. Survival curves comparing intracranial tumours split into classic and anaplastic groups and those comparing intracranial tumours with and without gain of 1q showed clear differences (Figures 3

and 4

**Figure 4:**
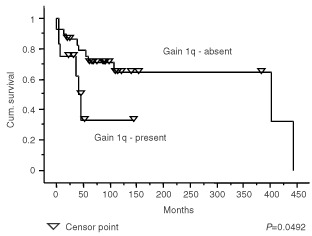
Kaplan–Meier plots showing a significant difference (*P*=0.0492) in the survival of patients with intracranial ependymomas with and without gain of 1q.

), and the difference (Figure 5

**Figure 5:**
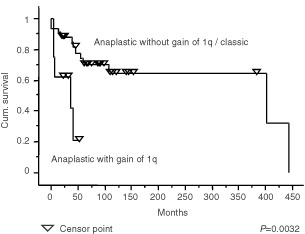
Kaplan–Meier plots showing a significant difference (*P*=0.0032) in the survival of patients with intracranial anaplastic ependymomas with gain of 1q and patients with intracranial classic ependymomas or anaplastic ependymomas without gain of 1q.

) between patients with anaplastic ependymomas showing gain of 1q and others with intracranial tumours was even more significant (*P*=0.0032). However, when the effect of multiple variables on the survival of patients with posterior fossa tumours was analysed (Cox proportional hazard ratios), trends towards a poor outcome were seen for children rather than adults, presence of metastases, no adjuvant therapy, and subtotal surgical resection in multivariate analyses (Table 5

**Table 5:**
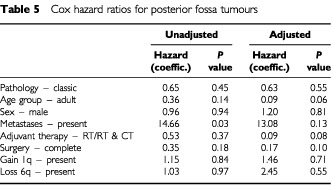
Cox hazard ratios for posterior fossa tumours

), but none of these effects reached statistical significance.

## DISCUSSION

This is the largest CGH study of ependymomas to be reported so far, and demonstrates that particular genetic profiles in these tumours reflect the principal division in their biology; specifically, that classic and anaplastic ependymomas occur mainly in the posterior fossa of children and young adults, and the myxopapillary variant occurs mainly in the region of the cauda equina in adults (Hamilton and Pollack, 1997; Ellison, 1998). In addition, we provide evidence to support the proposal that classic (WHO grade 2) ependymomas from the region of the spinal cord are genetically distinct from intracranial classic (grade 2) and anaplastic (grade 3) tumours (Ebert *et al*, 1999; Hirose *et al*, 2001).

Many previous cytogenetic or CGH studies of ependymomas have targeted tumours in children, and this may account for the paucity of genetic data on myxopapillary ependymomas; only one child from our series, a boy aged 15 years, presented with a myxopapillary spinal tumour. The consensus from previous studies is that about 40% of childhood ependymomas show no chromosomal imbalances (Reardon *et al*, 1999; Ward *et al*, 2001). This mirrors our data, which show a balanced chromosomal profile in 41% of childhood ependymomas, in contrast to only 9% of adult tumours. In this respect, a distinction is also seen between intracranial and spinal tumours; balanced profiles are evident in 32% of intracranial ependymomas, but in only one spinal tumour (3%). Another recent CGH study demonstrated balanced profiles in 21% of intracranial ependymomas, but none of the spinal tumours (Hirose *et al*, 2001). A common pattern of abnormalities across spinal (64%) and adult (56%) tumours in our study is gain across multiple whole chromosomes. This pattern suggests intermediate ploidy, and we confirmed this in a number of tumours using FISH. Such widespread imbalance was shown by the only myxopapillary ependymoma in one previous CGH study (Reardon *et al*, 1999), and by many spinal tumours in another (Hirose *et al*, 2001). Overall, the data indicate that spinal ependymomas, which present almost entirely in adult patients, and intracranial childhood tumours differ significantly in their genetic profiles. The former is characterised by widespread copy number aberrations, generally without evidence of rearrangement, and the latter by restricted specific gains or losses. The corollary of this is that low-grade tumours, which in practice are easier to treat, have more widespread abnormalities than the high-grade tumours, a situation that also pertains to neuroblastomas (Plantaz *et al*, 1997).

Loss of 22q has been the commonest abnormality in several genetic studies of ependymoma (Ransom *et al*, 1992; Mazewski *et al*, 1999; Vagner-Capodano *et al*, 1999), though gain of 1q or loss of 6q assumes predominance in others (Reardon *et al*, 1999; Ward *et al*, 2001). If the changes suggestive of intermediate ploidy are discounted, loss of 22q is the most frequent genetic abnormality in our series of ependymomas, occurring in 26% of ependymomas, which is in line with data from other CGH studies (Reardon *et al*, 1999; Hirose *et al*, 2001; Ward *et al*, 2001). However, the frequency of loss of 22q in ependymomas varies greatly (up to 71%) in previous studies that have used a variety of methods (Ransom *et al*, 1992; Neumann *et al*, 1993; Kramer *et al*, 1998; Vagner-Capodano *et al*, 1999; Zheng *et al*, 2000). This variability is likely to reflect ascertainment bias, because the frequency of loss of 22 in ependymomas varies according to histological variant, anatomic site, and age of the patient. The discrepancy between its occurrence in adults and children has been recorded in a large cytogenetic series (Mazewski *et al*, 1999), and several lines of evidence link loss of 22 to spinal ependymomas. There is an increasing susceptibility to gliomas in patients with neurofibromatosis type 2 (NF2), the gene for which, *NF2*, is at 22q12 (Wiestler *et al*, 2000). Most gliomas in NF2 (80%) are ependymomas, and three quarters of NF2 gliomas are spinal (Rodriguez and Berthrong, 1966). Mutations in the *NF2* gene are uncommon in sporadic ependymomas (von Haken *et al*, 1996), and appear to be restricted to spinal tumours. In a study of 62 ependymomas (Ebert *et al*, 1999), twelve showed allelic loss on 22q and six of them also had *NF2* mutations. All six ependymomas with mutations were classic variants from around the spinal cord.

Gain of 1q is a relatively frequent abnormality in ependymomas, and has been emphasised in CGH studies of paediatric tumours, where it has been reported in up to 22% of cases (Reardon *et al*, 1999). In our series, ependymomas with gain of 1q represent 17% of the total, but are significantly associated with childhood, posterior fossa location, and anaplastic histological features. Evidence for several extra copies of 1q is found in three of the tumours in our series, all anaplastic ependymomas from children. A similar phenomenon was reported in the study by Ward *et al* (2001), who also described three tumours with a combination of gain of 1q and loss of 16q as the only abnormalities. We found this combination once, in the anaplastic recurrence of a classic ependymoma that originally showed a balanced CGH profile. This combination strongly suggests a der(16)t(1;16), which is a well recognised secondary chromosomal change in a variety of tumours. This is the only instance of a change in genetic profile between two successive biopsies. Gain of 1q and loss of 10q as sole abnormalities occur twice in our cohort of intracranial tumours, both times in anaplastic ependymomas from children, and in one anaplastic tumour with other genetic abnormalities, which included amplification of 17q21qter. Further investigation is required to determine the significance of the association between gain of 1q and loss of 10q or loss of 16q in ependymomas. However, this phenomenon appears to occur in anaplastic childhood tumours with an aggressive behaviour. The association between gain of 1q and anaplastic ependymomas prompted us to look at the survival of patients with posterior fossa tumours divided according to histological variant and the presence of 1q. While numbers of tumours in the analysis are small, the designation of a tumour as anaplastic in the presence of gain of 1q was associated with a significantly worse survival curve. However, it is important to note that these variables were not significant prognostic indicators in a multivariate analysis, and this may reflect the need for a greater number of patients in such analyses.

Loss of 6q has been found in ependymomas with variable frequency (Reardon *et al*, 1999; Hirose *et al*, 2001; Ward *et al*, 2001). We demonstrate an overall frequency of 6%, matching the results of Ward *et al* (2001), but this is lower than results of up to 22% in some CGH/cytogenetic studies (Reardon *et al*, 1999). Again, the analysis of small numbers of patients, or of patients with tumours from a certain site, may account for this. For example, loss of 6q in our study attained its greatest frequency (26%) in adults with posterior fossa ependymomas, which was also a feature of another CGH study (Zheng *et al*, 2000).

Abnormalities of chromosomes 10 have featured prominently in the study of genetic abnormalities in gliomas. Loss of part or most of chromosome 10 is common in glioblastomas, and many of them also harbour mutations of the *PTEN* tumour suppressor gene at 10q23 (Bostrom *et al*, 1998; Duerr *et al*, 1998). Abnormalities of chromosome 10 appear less commonly in ependymomas, but among spinal tumours in our series clearly differentiate classic tumours from myxopapillary tumours. Microsatellite analysis has shown loss of 10q to be uncommon in ependymomas, and no mutation of the *PTEN* gene has been reported (Duerr *et al*, 1998; Ebert *et al*, 1999; Tong *et al*, 1999).

In summary, our study provides genetic data on a large range of ependymomas. We show that distinct genetic profiles characterise intracranial and spinal tumours, and tumours categorised by histological variant. In particular, we demonstrate that classic ependymomas from intracranial and spinal sites should be distinguished on the basis of genetic information. While our data suggest that gain of 1q is a possible marker for aggressive biological behaviour, further research is indicated to define clinically useful ways to incorporate histological and genetic assessments in the classification of ependymomas.
